# Neural Synchrony During Naturalistic Information Processing Is Associated With Aerobically Active Lifestyle and Cardiorespiratory Fitness in Cognitively Intact Older Adults

**DOI:** 10.3389/fnhum.2022.906099

**Published:** 2022-07-07

**Authors:** Tamir Eisenstein, Nir Giladi, Talma Hendler, Ofer Havakuk, Yulia Lerner

**Affiliations:** ^1^Sackler Faculty of Medicine, Tel Aviv University, Tel Aviv, Israel; ^2^Sagol Brain Institute, Tel Aviv Sourasky Medical Center, Tel Aviv, Israel; ^3^Department of Neurology, Tel Aviv Sourasky Medical Center, Tel Aviv, Israel; ^4^Sagol School of Neuroscience, Tel Aviv University, Tel Aviv, Israel; ^5^School of Psychological Sciences, Tel Aviv University, Tel Aviv, Israel; ^6^Department of Cardiology, Tel Aviv Sourasky Medical Center, Tel Aviv, Israel

**Keywords:** fMRI, neuroimaging, aerobic exercise, inter-subject correlation, real-life information

## Abstract

The functional neural mechanisms underlying the cognitive benefits of aerobic exercise have been a subject of ongoing research in recent years. However, while most neuroimaging studies to date which examined functional neural correlates of aerobic exercise have used simple stimuli in highly controlled and artificial experimental conditions, our everyday life experiences require a much more complex and dynamic neurocognitive processing. Therefore, we have used a naturalistic complex information processing fMRI paradigm of story comprehension to investigate the role of an aerobically active lifestyle in the processing of real-life cognitive-demanding situations. By employing the inter-subject correlation (inter-SC) approach, we have identified differences in reliable stimulus-induced neural responses between groups of aerobically active (*n* = 27) and non-active (*n* = 22) cognitively intact older adults (age 65–80). Since cardiorespiratory fitness has previously been suggested to play a key role in the neuroprotective potential of aerobic exercise, we have investigated its dose-response relationship with regional inter-subject neural responses. We found that aerobically active lifestyle and cardiorespiratory fitness were associated with more synchronized inter-subject neural responses during story comprehension in higher order cognitive and linguistic brain regions in the prefrontal and temporo-parietal cortices. In addition, while higher regional inter-SC values were associated with higher performance on a post-listening memory task, this was not translated to a significant between-group difference in task performance. We, therefore, suggest that the modulatory potential of aerobic exercise and cardiorespiratory fitness on cognitive processing may extend beyond simple and highly controlled stimuli to situations in which the brain faces continuous real-life complex information. Additional studies incorporating other aspects of real-life situations such as naturalistic visual stimuli, everyday life decision making, and motor responses in these situations are desired to further validate the observed relationship between aerobic exercise, cardiorespiratory fitness, and complex naturalistic information processing.

## Introduction

Aging is characterized by well-documented biological and functional manifestations (Boss and Seegmiller, 1981; Troen, 2003). One fundamental feature of aging is marked alterations in the structure and function of the brain, which are typically associated with some degree of cognitive performance decline, especially in higher-order cognitive functions such as processing of complex information, episodic memory, and executive functions (Stuss and Levine, 2002; Mattson and Magnus, 2006; Old and Naveh-Benjamin, 2008; Bishop et al., 2010; Grady, 2012). As the number of older adults suffering from cognitive decline is predicted to massively increase during the upcoming decades (Christensen et al., 2009; Alzheimer’s Association, 2021), identifying behavioral strategies through which age-related cognitive deterioration may be modified, are of primary importance to modern society.

Several lifestyle factors have been suggested to bare the potential to modify the trajectory of age-related cognitive function, including education, occupation, and leisure activities (Rovio et al., 2005; Sole-Padulles et al., 2009; Foubert-Samier et al., 2012). One such stimulus, aerobic exercise, has been linked with physiological and psychological health and well-being from childhood to late adulthood (Fiuza-Luces et al., 2013; Matta Mello Portugal et al., 2013). From an epidemiological perspective, an aerobically active lifestyle at different stages of the adult lifespan has been suggested to significantly reduce the risk of cognitive decline and dementia at older age (Rovio et al., 2005; Larson et al., 2006; Andel et al., 2008). However, the neural modifications that may underpin these behavioral effects are still a matter of intensive investigation. While previous studies of animal models have provided deep mechanistic insights into neuronal (van Praag et al., 1999, 2005), synaptic (Farmer et al., 2004), and neurovascular (Swain et al., 2003) adaptations that may result from engaging in aerobic physical activity, whether and how these findings may translate to humans is not clear.

Encouragingly, recent developments in non-invasive neuroimaging methods have enabled unprecedented views into the human brain *in vivo*. Using diverse neuroimaging modalities, previous studies have investigated neural correlates of aerobic exercise on both the structural and functional levels in the aging human brain. Interestingly, the findings arising from these studies have suggested that aerobic exercise may be more beneficial to brain regions that are more affected during the aging process such as the prefrontal cortex (Raz et al., 2005; Raz and Rodrigue, 2006), the default mode network (DMN; Salami et al., 2014; Palmqvist et al., 2017), and the hippocampus (Raz et al., 2004; Langnes et al., 2020). For example, aerobic exercise has been associated with greater volume of the prefrontal cortex in cognitively intact older adults (Colcombe et al., 2006), and the preservation of higher-order functions that are mediated by this region such as executive and control abilities (Colcombe and Kramer, 2003; Yogev-Seligmann et al., 2021). Furthermore, aerobically active lifestyle and aerobic exercise interventions in this age group have also been linked to higher micro- and macro-structural integrity of the hippocampus and preserved episodic memory performance (Erickson et al., 2011; Eisenstein et al., 2021a; Wilckens et al., 2021), although the effect of exercise on this region in humans is less consistent compared to that observed in animals (Wilckens et al., 2021).

In addition to brain morphology, aerobic exercise has also been linked to different aspects of brain function previously shown to be compromised in older adults such as task-related activation patterns (Nyberg et al., 2019) and resting-state intra- and inter-regional functional connectivity (Salami et al., 2014; Damoiseaux et al., 2016). For example, an aerobically active lifestyle has been related to less hippocampal hyperactivity during memory encoding and higher resting state functional coherence of the hippocampus with the DMN (Eisenstein et al., 2021b). Furthermore, increased functional connectivity of the DMN has also been demonstrated following a period of aerobic exercise intervention in cognitively healthy older adults (Voss et al., 2010). Interestingly, in many of the studies which had found a positive relationship between aerobic exercise and different brain aspects, a dose-response relationship was also found between those effects and peak oxygen consumption (Vo_2_peak), a well-known physiological correlate of aerobic/cardiorespiratory fitness (Balady et al., 2020). For example, Vo_2_peak was previously linked with hippocampal structure and function (Erickson et al., 2009, 2011; Eisenstein et al., 2021b), prefrontal function (Ishihara et al., 2020), and DMN functional connectivity in cognitively healthy older adults, making it a potential mediating factor of the neurocognitive effects of aerobic exercise.

While the relationship between aerobic exercise and brain function in older age has been given more and more attention in recent years, most functional neuroimaging studies which aimed to explore functional neural correlates of aerobic exercise and cardiorespiratory fitness have utilized relatively simple stimuli in controlled and artificial experimental environments (Smith et al., 2013; Hayes et al., 2017; Ishihara et al., 2020). However, this kind of cognitive paradigm may not reflect and capture the complex processing of continuous information that is embedded in everyday life situations. In contrast, naturalistic stimuli that mimic experiences from daily routine, such as listening to a spoken story, a lecture, or watching a movie, inherently require the integration of dynamic multidimensional information that spans from low-level sensory perception to complex higher-level linguistic and cognitive processing (Sonkusare et al., 2019). In addition, the comprehension of this kind of stimuli requires the ability to process a continuous flow of accumulated information over time as processing occurs at different time scales (e.g., seconds, minutes, and hours) and also across units of information (e.g., words, sentences, paragraphs, and narratives in the case of auditory stimuli; Xu et al., 2004).

Therefore, to address this gap in our understanding of the functional neural correlates of an aerobically active lifestyle and cardiorespiratory fitness in healthy older adults, the current study aimed to apply an inherently different approach. More specifically, we have investigated the neural responses to an ecological and complex auditory stimulus, i.e., an fMRI paradigm of a narrated story, in two groups of aerobically active and non-active cognitively intact older adults. In order to capture the dynamic nature of this kind of stimulus/paradigm, we have used a well-validated advanced fMRI-based analysis that measures the reliability of stimulus-evoked neural responses between participants, i.e., inter-subject correlation (inter-SC) analysis (Hasson et al., 2004; Lerner et al., 2011; Simony et al., 2016). Using inter-SC, we aimed to identify differences in neural processing of naturalistic stimuli between the aerobically active and non-active groups. The main strength of the inter-SC approach is that it increases the signal-to-noise (SNR) ratio in detecting stimulus-induced neural signals. This enhanced SNR is obtained from filtering out subject-specific intrinsic neural signals that may arise from cognitive processes unrelated to the processing of the ongoing stimulus or to subject-specific cognitive strategies. In addition, non-neuronal signals which arise from scanner-related and physiological artifacts, and can influence inter-regional correlations within a brain, will have much less effect on correlations across brains.

Inter-SC analysis has been recently used to demonstrate differences in neural responses between cognitively intact and cognitively declined older adults (patients with amnestic MCI, or aMCI) during the processing of a similar story comprehension paradigm (Yogev-Seligmann et al., 2016). It was found that cognitively intact older adults demonstrate higher neural synchrony in higher order cognitive and linguistic regions compared to the aMCI patients. In addition, we have recently demonstrated the potential of aerobic exercise intervention in modifying inter-subject neural synchrony in these patients (Yogev-Seligmann et al., 2021). Interestingly, increases in inter-SC following the aerobic training intervention were demonstrated mainly in brain regions that were previously shown to exhibit compromised inter-subject neural synchrony compared to cognitively healthy older adults (Yogev-Seligmann et al., 2016).

It is unclear, however, whether this kind of aerobic exercise-related advantages may be observed in older adults without a clinical cognitive deterioration. Therefore, the main aim of the current study was to investigate whether the relationship between an aerobically active lifestyle and brain function may be extended to the processing of complex naturalistic cognitive demands, such as the comprehension of a narrated story. Based on our previous findings in patients with aMCI (Yogev-Seligmann et al., 2021), we hypothesized that an aerobically active lifestyle will be associated with higher inter-subject neural synchrony (as measured with inter-SC), particularly in higher order cognitive and language brain regions. Next, we also aimed to investigate whether Vo2peak may be positively correlated with regional inter-SC values, based on its previously suggested mediating role of the neurocognitive effects of aerobic exercise. Lastly, we hypothesized that higher inter-SC values will be associated with task-related cognitive function, as expressed by a post-scanning cognitive evaluation.

## Materials and Methods

### Participants

Forty-nine older adults aged 65–80 were recruited for the study (21 women/28 men). All participants were recruited from the community *via* an online advert on the Israeli Ministry of Health research website, social media, and by “word-of-mouth”. Participants were fluent Hebrew speakers and reported no current or previous neuropsychiatric disorders (e.g., Parkinson’s disease, brain tumor, head injury, transient memory loss, stroke, psychosis, bipolar disorder, and depressive symptoms) including subjective cognitive/memory complaints, or any other current significant uncontrolled and unbalanced medical illness (e.g., cardiac or vascular disease, hypertension, type II diabetes, cancer, and autoimmune syndromes). The research was approved by the Human Studies Committee of Tel Aviv Sourasky Medical Center (TASMC), and all participants provided written informed consent to participate in the study.

### Aerobic Activity Lifestyle Habits Assessment

Participants’ current aerobically active lifestyle was assessed using a background interview that examined background characteristics as well as the physical exercise habits of the participants during the last year. The interview was administered face-to-face on the assessment day. Specifically, participants were asked about the amount of weekly exercise-oriented activity sessions of any type of aerobic exercise (i.e., walking, running, cycling, swimming, elliptical/cross training gym machines, etc.), or *“How often during the week in the passing year did you participate in leisure time aerobic activity for the purpose of sporting or exercise that lasted at least 20 min* *per session?”*. Next, based on the reported activity participants were divided into two groups, active (*n* = 27) and non-active (*n* = 22). A frequency of twice a week or higher was set as the cut-off for an active lifestyle based on previous methodologies (Rovio et al., 2005, 2010), findings (Liu-Ambrose et al., 2012), and recommendations (Voelcker-Rehage et al., 2010; Petersen et al., 2018). In addition, since we have used a subjective measure of active lifestyle, using a cut-off of several times per week may help to decrease potential information bias from participants’ reports compared to a cut-off of any aerobic activity at all.

### Vo_2_Peak Measurement

Forty-one participants (23 from the active group and 18 from the non-active group) of the total 49 participants also underwent a graded maximal cardiopulmonary exercise test performed on a cycle ergometer (Ergoselect 100, Ergoline, GmBH, Germany) to evaluate Vo_2_peak (Balady et al., 2020). Assessments were conducted at the Non-Invasive Cardiology Outpatient Clinic at TASMC. Tests were performed using a metabolic cart (ZAN, nSpire Health Inc., Longmont, CO) while continuously measuring breath-by-breath minute ventilation, carbon dioxide production (Vco_2_), oxygen consumption (Vo_2_), and respiratory exchange ratio (RER). In addition, a 12-lead electrocardiograph and non-invasive arterial saturation were monitored continuously as well as heart rate and blood pressure. All tests were supervised by a cardiologist and an exercise physiologist. An automated computerized ramp protocol was used to increase exercise intensity by 10 Watt/min for women and 15 Watt/min for men, while participants were asked to maintain a constant velocity of 60 revolutions per minute. All tests were performed until volitional exhaustion, and no adverse events or medical symptoms were reported. RER value ≥ 1.1 was used as the indication for a satisfactory effort level during the test (Balady et al., 2020) and was demonstrated in all examinations. Plateau in Vo_2_ was not evident in any participant, therefore the highest Vo_2_ demonstrated in each test was considered as a Vo_2_peak. The highest average Vo_2_ value recorded during an intensity interval (2/2.5 watts increment for women and men, respectively) was considered as the Vo_2_peak value obtained from the procedure and that was used in further analyses.

### Complex Information Processing Paradigm

This paradigm has been described in detail in a previous study (Yogev-Seligmann et al., 2016). Briefly, the participants were asked to listen to a real-life plotted 8-min story told in Hebrew by a professional storyteller and recorded especially for the study, with no visual stimulus presented in parallel. Attentive listening to the story was confirmed by informing the participants that after the scanning, they will be referred to the story and tested on its content. An inter-subject correlation (inter-SC) analysis (see below) was used to identify and compare reliable neural responses during the story within and between the study groups (Hasson et al., 2004; Lerner et al., 2011; Yogev-Seligmann et al., 2016, 2021). Following the acquisition phase in the scanner, we performed a post-listening memory test, assessing the accuracy of recollecting the story details (Simony et al., 2016). In the test, participants were asked to perform a “Fill-in-the-blank” task, in which the full auditory narrative of the story was presented as a written version with missing words which the participants were asked to fill. Importantly, the words that needed to be filled were all pieces of information that could not have been “guessed” correctly just based on the context of the other words in the sentence. Each correct word was graded with 1 point. A context-related word which was not identical to the original piece of information was graded with 0.5 points (e.g., “Fruits” instead of “Bananas”). Overall, 41 missing words were presented in the test.

### MRI Acquisition

MRI scanning was performed at TASMC on a 3 T Siemens system (MAGNETOM Prisma, Germany). High resolution, anatomical T1-weighted images (voxel size = 1 × 1 × 1 mm) were acquired with a magnetization prepared rapid acquisition gradient-echo protocols with 176 contiguous slices using the following parameters: field of view (FOV) = 256 mm; matrix size = 256 × 192; repetition time (TR) = 1,740 ms; echo time (TE) = 2.74 ms, inversion time (TI) = 976 ms, flip angle (FA) = 8°. These anatomical volumes were used for co-registration with functional images. Blood oxygenation level dependent (BOLD) functional MRI was acquired with T2*-weighted imaging using the following parameters: TR = 1,500 ms (overall 320 TRs); TE = 30 ms; FA = 75°; FOV = 22 × 22 cm^2^; matrix size = 64 × 64; 27 slices of 3 mm thickness, 1 mm gap. The slices were positioned nearly horizontal to cover the entire temporal lobe and the parts of the frontal lobe that are involved in hearing and language processing, as well as nearly all the occipital and parietal lobes. To minimize head movements, participants’ heads were stabilized with foam padding. MRI-compatible headphones (OPTOACTIVEtm) were used to considerably attenuate the scanner noise and communicate with the participants during the session.

### MRI Data Analysis

fMRI data were analyzed with the BrainVoyager QX 2.8 software package (Brain Innovation, Maastricht, The Netherlands) and with in-house software written in MATLAB (The MathWorks). Preprocessing of the functional scans included slice time and motion correction, linear trend removal, high-pass filtering (cut-off: 0.01 Hz), spatial smoothing using a 6 mm full-width at half-maximum kernel, and cropping of the first five TRs to eliminate preprocessing artifacts and to allow the hemodynamic responses to reach a steady state. Data analysis was performed separately for each participant. The functional images were co-registered with the anatomical images using a two-step, semi-automatic procedure. First, an initial alignment by BrainVoyager was performed. Next, a subsequent advanced manual alignment was applied. Finally, the data were incorporated into the three-dimensional data sets through trilinear interpolation. The complete functional dataset was transformed into a common 3D Talairach space (Talairach and Tournoux, 1998). No participants were excluded due to excessive head motion (greater than 2 mm). All analyses were conducted using a whole-brain approach. Next, neural responses during information processing were analyzed using inter-SC analysis (Hasson et al., 2004; Lerner et al., 2011; Yogev-Seligmann et al., 2016, 2021). This approach explores to what extent similar brain regions of different participants show synchronization of neural responses to natural stimuli. This synchronization is exhibited by the significant correlation between each participant’s neural response and the other participants’ responses, indicating response reliability. Inter-SC maps were constructed voxel-by-voxel in Talairach space for each group by comparing the fMRI response time-courses across participants. First, the Pearson product-moment correlation was computed between a voxel’s fMRI time-course in one individual and the average of that voxel’s fMRI time-courses in the remaining participants. Next, the average correlation was calculated at every voxel. The statistical significance of the inter-SC analyses was assessed using a phase-randomization procedure. Phase-randomization was performed by applying a fast Fourier transform to the signal, randomizing the phase of each Fourier component, and then inverting the Fourier transformation. Thus, the power spectrum was preserved but the correlation between any pair of such phase-randomized time-courses had an expected value of 0. Phase-randomized time-courses were generated for every measured fMRI time-course from every voxel in each participant. A correlation value was then computed (as detailed above) for every voxel. This process was repeated 5,000 times to generate a null distribution of the correlation values, separately for each voxel. Statistical significance was assessed by comparing empirical correlation values (without phase randomization) to these null distributions. The Benjamini–Hochberg–Yekutieli false-discovery procedure, which controls the false discovery rate (FDR) under assumptions of dependence, was used to correct for multiple comparisons (Benjamini and Hochberg, 1995; Benjamini and Yekutieli, 2001; Genovese et al., 2002). The differences in neural response reliability between groups were assessed using a 2-tailed *t*-test.

### General Cognitive Evaluation

All participants were evaluated for general cognitive functioning and were screened for objective general cognitive decline using the Montreal Cognitive Assessment (MoCA; Oren et al., 2015).

### Statistical Analysis

Statistical analyses and visualizations were performed and constructed with R v4.1.2^1^. To assess the relationship between Vo2peak, inter-SC values, and the story test score we have computed partial Pearson correlations controlling for age, sex, and years of education. Between-group comparisons of continuous variables (age, years of education, Vo_2_peak, weekly days of exercise, story scores, and MoCA) were assessed using an independent samples t-test. Between-group differences in the proportions between males and females, marital status, smoking status, and hypertension diagnosis were assessed using the Chi-squared test. As only two participants reported being currently smoking, we characterized the participants as “*current or past smokers*” and “*non-smokers*”. One-tailed between-group independent correlation coefficients comparison was conducted to explore between-group differences in the strength of the relationship between Vo_2_peak and inter-SC values in the regions identified in the fMRI between-group analysis. This was conducted with the Fisher’s r-to-z transformation using the “*cocor*” package implemented in R.

## Results

### Study Participants

The active and non-active groups were not statistically different in any of the background characteristics, including age, sex, education, and general cognitive functioning (*p* > 0.05; Table 1).

**Table 1:** Background characteristics of the participants (means ± SD).

**Variable**	**Whole sample** **(*n* = 49)**	**Aerobically active** **(*n* = 27)**	**Non-active** **(*n* = 22)**	**Between-group *p*-value**
Age (years)	70.98 ± 3.9	70.41 ± 4.2	71.68 ± 3.6	0.267
Sex (female/male)	21/28	10/17	11/11	0.362
Education (years)	15.90 ± 3.4	16.26 ± 3.8	15.45 ± 2.9	0.415
Marital status (married/ unmarried)	43/6	25/2	18/4	0.252
Smoking (current or past smokers/non-smokers)	21/26	11/15	10/11	0.926
Hypertension (diagnosed/undiagnosed)	8/41	4/23	4/19	0.751
MoCA (raw score)	24.93 ± 2.5	25.17 ± 2.2	24.59 ± 2.8	0.463

*MoCA, Montreal Cognitive Assessment; SD, standard deviation*.

### Aerobic Exercise Habits and Vo_2_Peak Characteristics

The participants’ report of weekly aerobic activity ranged from being completely sedentary (not engaging in exercise-oriented activities, *n* = 17) to 6 days per week (*n* = 3). Seventeen participants reported exercising 3 times per week. Exercising on a single day (*n* = 5), twice a week (*n* = 3), four times per week (*n* = 3), and five times (*n* = 1) were also reported. The active group demonstrated a significantly higher weekly frequency of exercise sessions and Vo_2_peak values (Table 2).

**Table 2:** Aerobic activity and fitness characteristics of the participants (means ± SD).

**Variable**	**Whole sample**	**Aerobically active**	**Non-active**	**Between-group *p*-value**
Aerobic exercise (d/week)	1.98 ± 1.8 (*n* = 49)	2.85 ± 1.6 (*n* = 27)	0.91 ± 1.5 (*n* = 22)	**<0.001**
Vo_2_peak (ml/kg/min)	25.37 ± 8.3 (*n* = 42)	30.60 ± 7.2 (*n* = 23)	19.04 ± 3.8 (*n* = 19)	**<0.001**

*d/week, days per week; kg, kilogram; min, minute; ml, milliliter; SD, standard deviation; Vo_2_peak, peak oxygen consumption. Values in bold: statistically significant*.

### Between-Group Differences in Neural Topology of Complex Information Processing

**Figure 1:**
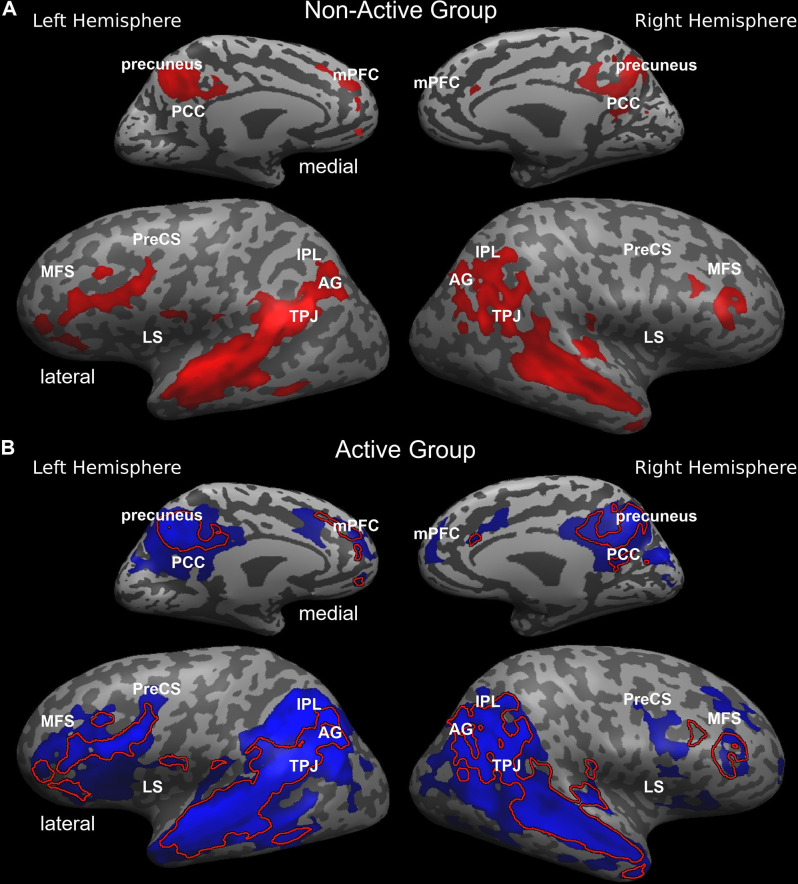
Inter-SC topography during complex information processing in the non-active (**A**, red) and active (**B**; blue) groups. Inter-SC maps of the non-active group are overlaid in **(B)** and represented with a red line. AG, angular gyrus; IPL, inferior parietal lobule; LS, lateral sulcus/insular cortex; MCC, middle cingulate cortex; MFS, middle frontal sulcus; mPFC, medial prefrontal cortex; PCC, posterior cingulate cortex; PreCS, precentral sulcus; TPJ, temporoparietal junction.

**Figure 2:**
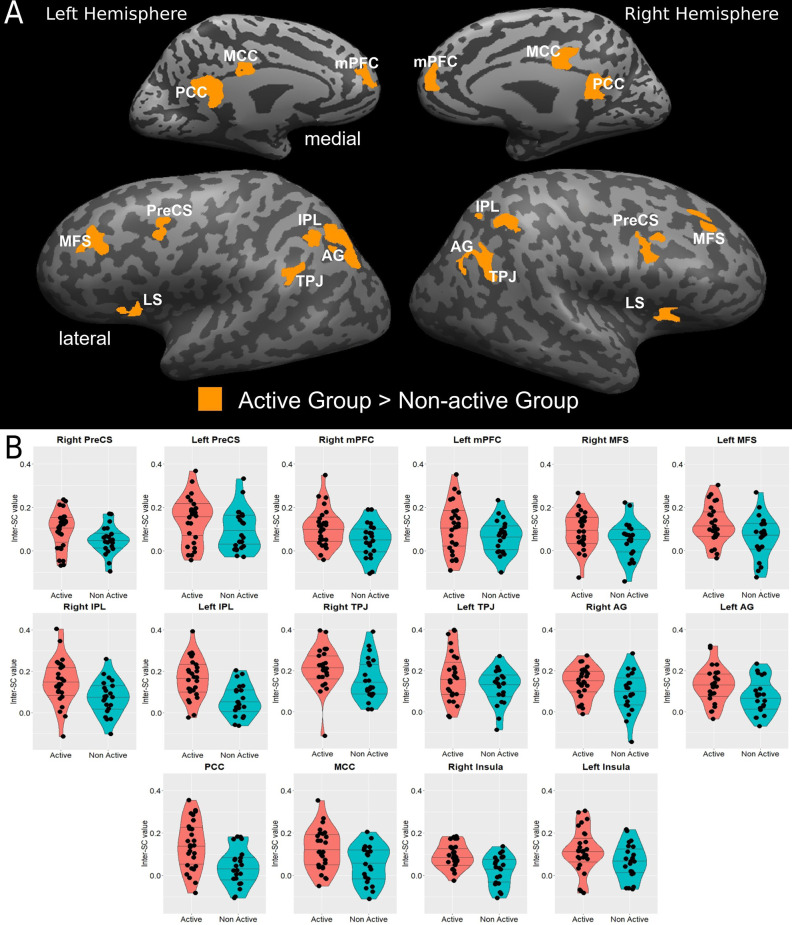
**(A)** Statistical brain map of between-group differences in inter-SCs during complex information processing. **(B)** Violin plots of between-group differences in the inter-SC values in significantly different regions are presented in **(B)**; horizontal lines represent 25%, 50%, and 75% quantile. AG, angular gyrus; IPL, inferior parietal lobule; LS, lateral sulcus/insular cortex; MCC, middle cingulate cortex; MFS, middle frontal sulcus; mPFC, medial prefrontal cortex; PCC, posterior cingulate cortex; PreCS, precentral sulcus; TPJ, temporoparietal junction.

Both groups employ the same neural system which interprets and processes story information. Specifically, both inter-SC maps included regions that spanned from the early auditory processing areas to higher order language and cognitive regions (Figure 1), and were similar to previously demonstrated response organization in healthy older adults during naturalistic story comprehension (Yogev-Seligmann et al., 2016). Moreover, the cortical regions that demonstrated reliable responses in the non-active group completely overlapped with the regions observed in the active group. However, the coherency of neural responses became more differentiated in higher-order regions as the active group demonstrated more reliable inter-subject responses in lateral and medial prefrontal cortices, posteromedial, and lateral parietal regions. More specifically, between group t-test revealed that the active older adults demonstrated significantly higher synchronized responses and inter-SC in the middle frontal sulcus (MFS), precentral sulcus (PreCS), medial prefrontal cortex (mPFC), angular gyrus (AG), inferior parietal lobule (IPL), temporoparietal junction (TPJ), and insula, bilaterally, and in the middle and posterior cingulate cortices (MCC and PCC, respectively; Figure 2). Table 3 provides Talairach coordinates for the noted regions.

**Table 3:** Talairach coordinates of the ROIs revealing significant differences in between-group comparison.

**Brain region**	BA	Talairach coordinates			Cluster size
		x	y	z	
Frontal Lobe
L. Middle frontal sulcus (MFS)	9	−34	38	26	870
R. Middle frontal sulcus (MFS)	9	28	32	34	600
L. Precentral Sulcus (PreCS)	9	−39	3	35	700
R. Precentral Sulcus (PreCS)	9	41	8	31	830
L. Medial Prefrontal Cortex (mPFC)	6	−7	43	33	560
R. Medial Prefrontal Cortex (mPFC)	10	7	56	19	520
Parietal Lobe
L. Angular Gyrus (AG)	39	−41	−61	34	970
R. Angular Gyrus (AG)	39	43	−59	32	920
L. Inferior parietal lobule (IPL)	40	−39	−51	38	360
R. Inferior parietal lobule (IPL)	40	40	−50	42	530
Temporal Lobe
L. Temporo-parietal junction (TPJ)	39	−50	−58	28	330
R. Temporo-parietal junction (TPJ)	39	52	−53	21	420
Insular Cortex
L. Insula	13	−30	15	12	950
R. Insula	13	31	21	6	680
Cingulate Cortex
Posterior Cingulate (PCC)	29	0	−49	11	920
Middle Cingulate (MCC)	31	1	−27	35	760

*BA, Brodmann area; L, left; R, right*.

### Relationship Between Vo_2_Peak and Regional Inter-SC

**Figure 3:**
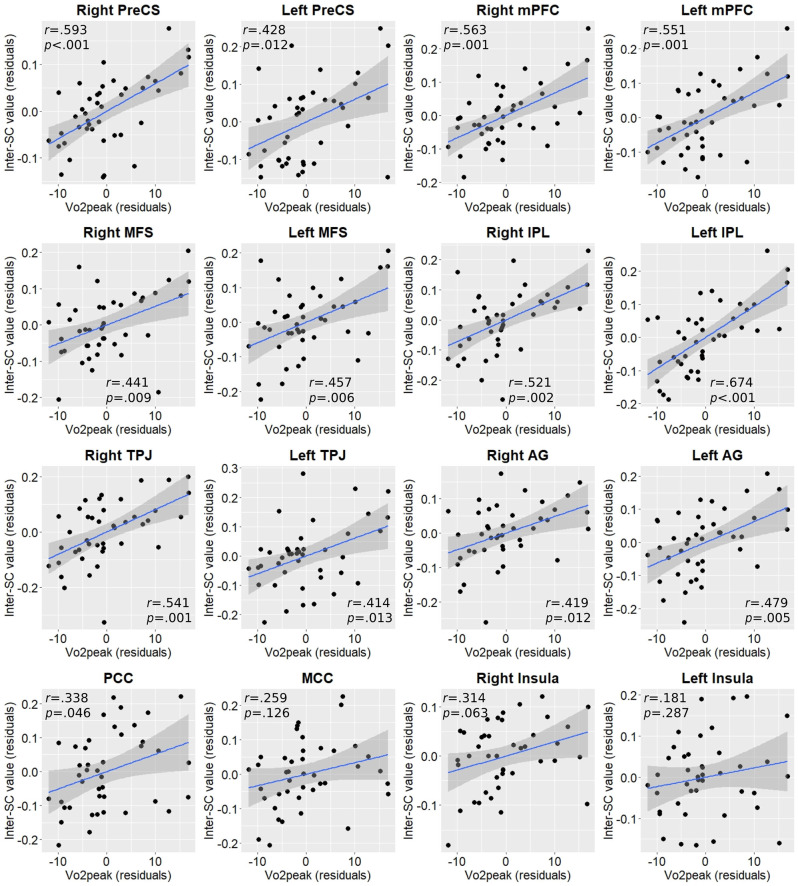
Partial correlation plots between Vo_2_peak and inter-SC values in regions exhibiting between-group differences. P-values are FDR-corrected for multiple comparisons. AG, angular gyrus; IPL, inferior parietal lobule; MCC, middle cingulate cortex; MFS, middle frontal sulcus; mPFC, medial prefrontal cortex; PCC, posterior cingulate cortex; PreCS, precentral sulcus; TPJ, temporoparietal junction.

We then measured the dose-response relationship between Vo_2_peak and inter-SC values across the whole sample in the regions identified in the between-group analysis, by computing the partial correlation controlling for age, sex, and years of education. Almost all regional inter-SC values demonstrated statistically significant moderate positive correlations with Vo_2_peak values (Figure 3).

Interestingly, when we tested these correlations within each group, most correlations remain significant in the active group but not in the non-active group (Supplementary Table 1), although this should be addressed with caution as the non-active group is smaller and, hence, lower powered. A direct comparison between the correlation coefficients of the two groups in each region showed that the active group demonstrated a statistically significant higher correlation only in the left IPL (*Z* = 2.13, *p* = 0.017).

### Relationship Between Regional Inter-SC and Cognitive Performance

**Figure 4:**
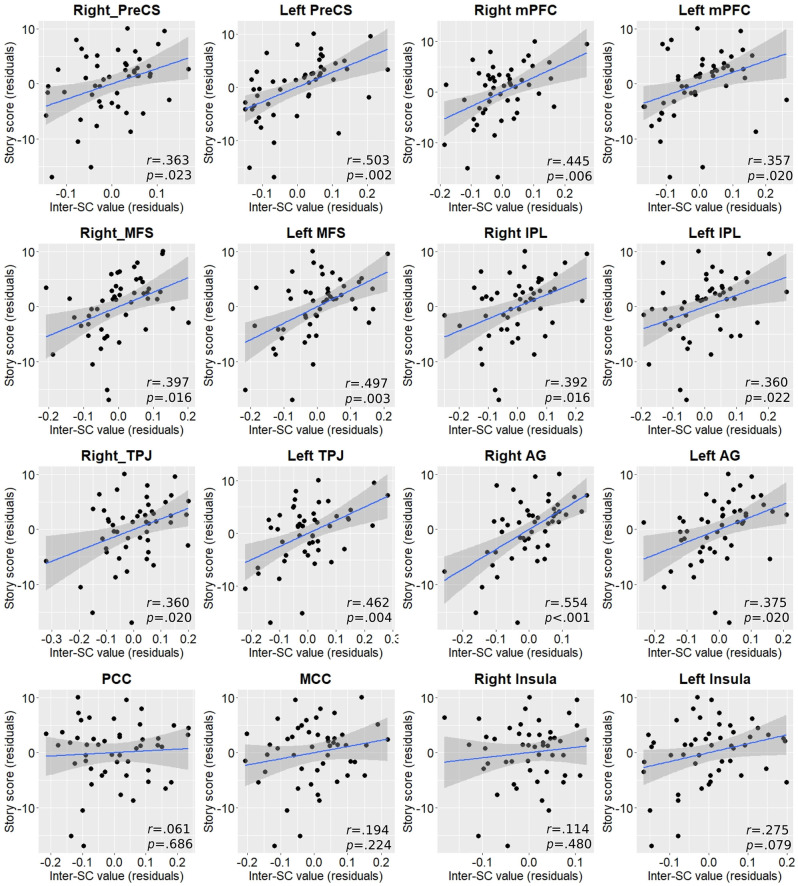
Partial correlation plots between inter-SC values and post-scan story score in regions exhibiting between-group differences. P-values are FDR-corrected for multiple comparisons. AG, angular gyrus; IPL, inferior parietal lobule; MCC, middle cingulate cortex; MFS, middle frontal sulcus; mPFC, medial prefrontal cortex; PCC, posterior cingulate cortex; PreCS, precentral sulcus; TPJ, temporoparietal junction.

To assess the relationship between each region’s inter-SC and post-scanning memory score we again computed partial correlations controlling for age, sex, and years of education. As with Vo_2_peak, almost in all regions inter-SC values demonstrated statistically significant moderate positive correlations with higher cognitive performance and memory scores (Figure 4).

### Relationship Between Aerobic Activity, Vo_2_Peak, and Post Scanning Cognitive Performance

**Figure 5:**
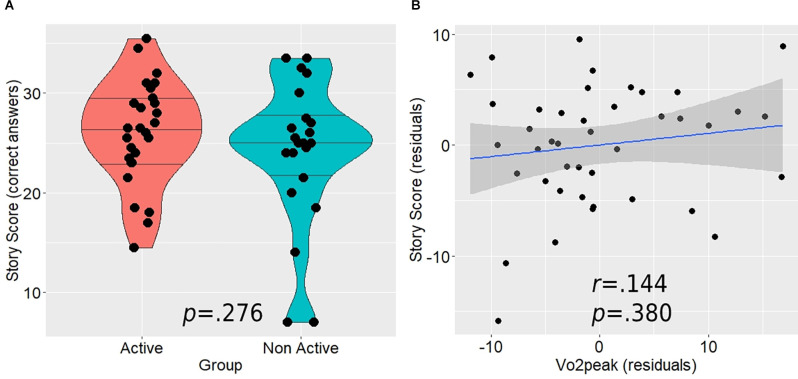
The relationship between the post-scan story score, aerobic activity level **(A)**, and Vo_2_peak **(B)**. Horizontal lines in between group violin plot **(A)** represent 25%, 50%, and 75% quantiles.

Although the active group demonstrated higher mean performance on the post-scanning memory test (26.04 ± 5.2 vs. 24.07 ± 7.3), this difference was not statistically significant (*t*_(47)_ = 1.103, *p* = 0.276; Figure 5A). In addition, the correlation between Vo_2_peak and the memory scores was relatively weak and non-significant as well (*r*_(37)_ = 0.144, *p* = 0.380; Figure 5B). When we tested this relationship separately within each group, we found generally similar correlations (active: *r*_(18)_ = 0.224, *p* = 0.342; ; non-active: *r*_(14)_ = 0.276, *p* = 0.300).

## Discussion

The current study aimed to investigate the relationship between an aerobically active lifestyle and functional neural correlates of complex naturalistic information processing (i.e., a narrated story) in older adults without an objective or subjective cognitive decline. To this end, we have used an inter-SC analysis to examine between-group differences in inter-subject neural synchrony during a story comprehension fMRI paradigm (Hasson et al., 2004; Lerner et al., 2011). In addition, we aimed to examine whether Vo_2_peak, a well-known measure of cardiorespiratory fitness (Balady et al., 2020), may demonstrate a positive dose-response relationship with regional inter-SC values across the groups and the whole sample.

We, first, found that both groups demonstrated a topographical pattern of inter-subject neural responses which was generally similar to that previously observed in older and young adults during the processing of auditory real-life information (Lerner et al., 2011; Yogev-Seligmann et al., 2016). However, despite this general similarity, we did find differences in synchronized inter-subject neural responses between an aerobically active and non-active older adults, especially in higher-order cognitive and linguistic brain regions (Friederici, 2012; Hertrich et al., 2020). More specifically, we found active older adults to demonstrate higher inter-SC values mainly in the frontal and temporo-parietal regions, with most of these areas corresponding to the topography of the DMN, including the medial prefrontal cortex and the medial and inferior lateral parietal cortices. In addition, we found Vo_2_peak to exhibit a positive dose-response relationship with the inter-SC values at the subject level in most of the regions in which an active lifestyle was associated with more synchronized responses. Lastly, we found higher inter-SC to positively correlate with performance in the post-listening memory task of story recollection, although this was not translated to a statistically significant difference in performance between the groups.

Interestingly, most of the brain regions observed in the current study to demonstrate higher inter-SC in aerobically active older adults were also previously found to show increased inter-SC during story comprehension following an aerobic exercise intervention in patients with aMCI (Yogev-Seligmann et al., 2021). This in turn suggests that these findings may indeed represent an expression of aerobic exercise-related modification of functional brain processing. This possibility is further supported by our recent work in aMCI in which a non-aerobic physical training did not elicit a similar regional pattern of increased inter-SC, and also by previous studies which had linked aerobic exercise/cardiorespiratory fitness with frontal and DMN function. The prefrontal cortex is perhaps the brain region which demonstrates the most consistent positive relationship between aerobic exercise and cardiorespiratory fitness. Higher aerobic fitness was previously linked with increased activation of executive-control prefrontal regions during memory tasks in both healthy and cognitively declined individuals (Eisenstein et al., 2020; Ishihara et al., 2020). In addition, functional neural modifications in similar prefrontal locations were observed in patients with aMCI across different cognitive tasks following aerobic exercise intervention, which also yielded significant improvements in Vo_2_peak (Yogev-Seligmann et al., 2021). The prefrontal cortex is also inherently implicated in the functional neuroanatomy of the DMN, in particular its medial surface (Andrews-Hanna et al., 2010). Higher levels of aerobic activity and cardiorespiratory fitness were also recently shown to be associated with higher functional connectivity of the core hubs of the DMN, namely the medial prefrontal and medial parietal cortices, in cognitively intact older adults (Eisenstein et al., 2021b). Those previous findings support the results of the current study in characterizing the neuroprotective modulating potential of aerobic exercise and fitness on brain function, specifically in these areas. This exercise- and fitness-related modulation of DMN function is of particular interest and importance given the fact that compromised DMN function is a hallmark characteristic of the aging brain (Salami et al., 2014), and is also associated with early expression of Alzheimer’s disease pathology such as early amyloid-beta accumulation (Palmqvist et al., 2017). Moreover, our results complement previous findings which highlighted the potential key role that cardiorespiratory adaptations to aerobic exercise (in this case peak oxygen consumption) may subserve in the effects of this lifestyle-related behavior on brain structure and function (Erickson et al., 2011; Kleemeyer et al., 2015; Yogev-Seligmann et al., 2021).

The next question that should naturally be asked is whether the observed regional differences in inter-subject neural synchrony in the current study are cognitively meaningful? Naturalistic real-life situations demand the accumulation and integration of information over time. Previously, a set of higher-order brain regions were identified as playing a key part in these processes including the TPJ, mPFC, AG, PCC, and IPL (Lerner et al., 2011; Simony et al., 2016). These regions which are associated with the DMN (Andrews-Hanna et al., 2010) also support Theory of Mind (ToM) processing (Li et al., 2014). They have previously been shown to be modulated by the coherence of the temporal structure of the stimulus, and to demonstrate more synchronized responses with a longer temporal receptive window from auditory perception to full story comprehension, as required during our naturalistic cognitive paradigm (Lerner et al., 2011; Simony et al., 2016). Interestingly, these are also the main regions that were identified in the current study to demonstrate more synchronized neural responses in aerobically active older adults compared to non-active older adults during the story. Listening to a story, even short, is expected to elicit activation in the ToM network. Although the cortical areas which the active and non-active older adults were using to process and interpret the complex information largely overlapped, the important differences included regions that might be considered as a part of the mentalizing network but might also be considered as nodes engaged in ToM computations, or an attentional network (i.e., mPFC, middle cingulate, TPJ, AG/posterior STG; Menon, 2013). These regions may substantially modify performance and efficiency that is consistent with our findings for cognitive performance. The importance of these regions in the processing of complex real-life information is further highlighted by two aspects. First, in the current study, we found significant relationships between the extent of inter-SC values in these regions and the performance on the post-listening memory task, meaning that participants demonstrating higher level of inter-subject synchronized neural responses were more successfully able to recall information from the complex stimulus they were subjected to process. Second, in a recent study assessing age-related changes in the processing of integrated auditory and visual information (i.e., movie watching) an age-related decline in inter-subject synchronized responses was found in similar regions, namely medial and lateral PFC, inferior parietal regions, and posterior cingulate region (Geerligs and Campbell, 2018) . With that being said, we should note, however, that although the active group in the current study demonstrated higher mean performance on the post-scan memory task, neither between-group comparison of the task score, or a correlation analysis between this score and Vo_2_peak yielded a statistically significant effect.

### Study Limitations

The main limitation of the current study lies in its cross-sectional nature. Although previous evidence from animal models and human participants supports a modulatory potential of aerobic exercise on brain function, we cannot conclude any causal effects of an aerobically active lifestyle on the functional neural processes observed in the current study. These results may serve as a starting point for future longitudinal and interventional studies that may shed further light on the time-dependence relationship between aerobic exercise and complex information processing in the aging brain and to establish a causal effect. Another issue that should be kept in mind is that other factors that have not been evaluated in this study could potentially contribute to the explanation of the relationship observed. These include, but are not limited to, differences in genetic background and latent pathological processes. Although we excluded participants with unbalanced medical conditions, this older population is more prone to pathologies, and it is possible that at least some of the included participants had ongoing pathological processes that are still not symptomatically expressed and therefore they were not aware of.

## Conclusions

Our study provides additional evidence of the neuroprotective potential of aerobic exercise and cardiorespiratory fitness on brain function and cognitive processing in the aging human brain. As the brain relies on a hierarchical regional topography when processing complex information over time, we have demonstrated that the modulatory potential of aerobic exercise and fitness on cognitive processing may extend beyond simple stimuli in highly controlled and artificial experimental conditions to situations in which the brain faces real-life ongoing complex information. Additional studies incorporating other aspects of real-life-related situations such as processing of complex visual stimuli and even paradigms involving decision making and motor responses in real-life situations are required to further validate the observed relationship between aerobic exercise, cardiorespiratory fitness, and complex naturalistic information processing in the current study.

## Data Availability Statement

Due to medical confidentiality and since participants did not consent to having their data publicly published, the unidentified data (e.g., data spreadsheet) and code that support the findings of this study are available from the corresponding author, TE, without undue reservation.

## Ethics Statement

The studies involving human participants were reviewed and approved by Human Studies Committee of Tel Aviv Sourasky Medical Center. The patients/participants provided their written informed consent to participate in this study.

## Author Contributions

TE: conceptualization, investigation, methodology, software, formal analysis, writing—original draft, writing—review and editing, project administration, and visualization. NG: supervision, conceptualization, writing—review and editing. TH: resources and funding acquisition. OH: project administration. YL: conceptualization, methodology, supervision, writing—review and editing, project administration, resources, and funding acquisition. All authors contributed to the article and approved the submitted version.

## Conflict of Interest

The authors declare that the research was conducted in the absence of any commercial or financial relationships that could be construed as a potential conflict of interest.

## Publisher’s Note

All claims expressed in this article are solely those of the authors and do not necessarily represent those of their affiliated organizations, or those of the publisher, the editors and the reviewers. Any product that may be evaluated in this article, or claim that may be made by its manufacturer, is not guaranteed or endorsed by the publisher.

## Footnotes

^1^ https://www.R-project.org/
