# Supplementary material for: Neural Synchrony During Naturalistic Information Processing Is Associated With Aerobically Active Lifestyle and Cardiorespiratory Fitness in Cognitively Intact Older Adults
Source: Front Hum Neurosci. 2022 Jul 7;16:906099. doi: 10.3389/fnhum.2022.906099 (PMC9300901; doi:10.3389/fnhum.2022.906099)
Supplement: Supplementary file 1 [file Table_1.docx]

Supplementary Table 1. Within-group relationship between Vo2peak and inter-SC. P-values are corrected for multiple comparisons in each group separately.

| Region | Aerobically active | Non-active |
| --- | --- | --- |
| R PreCS | *r*(18) = .604  *p* = .027 | *r*(14) = .619  *p* = .176 |
| L PreCS | *r*(18) = .463  *p* = .058 | *r*(14) = .370  *p* = .361 |
| R mPFC | *r*(18) = .586  *p* = .022 | *r*(14) = .443  *p* = .344 |
| L mPFC | *r*(18) = .699  *p* = .005 | *r*(14) = .442  *p* = .275 |
| R MFS | *r*(18) = .582  *p* = .019 | *r*(14) = .172  *p* = .764 |
| L MFS | *r*(18) = .550  *p* = .019 | *r*(14) = .223  *p* = .722 |
| R IPL | *r*(18) = .604  *p* = .020 | *r*(14) = .148  *p* = .667 |
| L IPL | *r*(18) = .764  *p* = .001 | *r*(14) = .158  *p* = .687 |
| R TPJ | *r*(18) = .561  *p* = .018 | *r*(14) = .517  *p* = .320 |
| L TPJ | *r*(18) = .415  *p* = .092 | *r*(14) = .380  *p* = .392 |
| R AG | *r*(18) = .563  *p* = .020 | *r*(14) = .449  *p* = .432 |
| L AG | *r*(18) = .582  *p* = .016 | *r*(14) = .202  *p* = .726 |
| PCC | *r*(18) = .026  *p* = .914 | *r*(14) = .170  *p* = .704 |
| MCC | *r*(18) = -.176  *p* = .564 | *r*(14) = .106  *p* = .742 |
| R Insula | *r*(18) = -.140  *p* = .635 | *r*(14) = .051  *p* = .850 |
| L Insula | *r*(18) = .049  *p* = .893 | *r*(14) = .242  *p* = .734 |

R = right; L = left; AG = angular gyrus; IPL = inferior parietal lobule; MCC = middle cingulate cortex; MFS = middle frontal sulcus; mPFC = medial prefrontal cortex; PCC = posterior cingulate cortex; PreCS = precentral sulcus; TPJ = temporoparietal junction
